# PI3K mediated activation of GSK-3β reduces at-level primary afferent growth responses associated with excitotoxic spinal cord injury dysesthesias

**DOI:** 10.1186/s12990-015-0041-2

**Published:** 2015-06-21

**Authors:** Sonja K Bareiss, Elizabeth Dugan, Kori L Brewer

**Affiliations:** Department of Physical Therapy, College of Allied Health Sciences, East Carolina University, 600 Moye Blvd., Room 2405E, Greenville, NC 27834 USA; Department of Emergency Medicine, East Carolina University, 600 Moye Blvd., Room 2405E, Greenville, NC 27834 USA

**Keywords:** GSK-3β, Spinal cord injury (SCI), Pain, Dysesthesias, Dorsal root ganglia (DRG), Neurite outgrowth

## Abstract

**Background:**

Neuropathic pain and sensory abnormalities are a debilitating secondary consequence of spinal cord injury (SCI). Maladaptive structural plasticity is gaining recognition for its role in contributing to the development of post SCI pain syndromes. We previously demonstrated that excitotoxic induced SCI dysesthesias are associated with enhanced dorsal root ganglia (DRG) neuronal outgrowth. Although glycogen synthase kinase-3β (GSK-3β) is a known intracellular regulator neuronal growth, the potential contribution to primary afferent growth responses following SCI are undefined. We hypothesized that SCI triggers inhibition of GSK-3β signaling resulting in enhanced DRG growth responses, and that PI3K mediated activation of GSK-3β can prevent this growth and the development of at-level pain syndromes.

**Results:**

Excitotoxic SCI using intraspinal quisqualic acid (QUIS) resulted in inhibition of GSK-3β in the superficial spinal cord dorsal horn and adjacent DRG. Double immunofluorescent staining showed that GSK-3β^P^ was expressed in DRG neurons, especially small nociceptive, CGRP and IB4-positive neurons. Intrathecal administration of a potent PI3-kinase inhibitor (LY294002), a known GSK-3β activator, significantly decreased GSK-3β^P^ expression levels in the dorsal horn. QUIS injection resulted in early (3 days) and sustained (14 days) DRG neurite outgrowth of small and subsequently large fibers that was reduced with short term (3 days) administration of LY294002. Furthermore, LY294002 treatment initiated on the date of injury, prevented the development of overgrooming, a spontaneous at-level pain related dysesthesia.

**Conclusions:**

QUIS induced SCI resulted in inhibition of GSK-3β in primary afferents and enhanced at-level DRG intrinsic growth (neurite elongation and initiation). Early PI3K mediated activation of GSK-3β attenuated QUIS-induced DRG neurite outgrowth and prevented the development of at-level dysesthesias.

## Background

Chronic neuropathic pain and sensory abnormalities are common secondary consequences of spinal cord injury (SCI), affecting >60% of patients with traumatic or ischemic injury [1–4], and effective treatments for this pain remain elusive. Clinically, SCI pain and associated dysesthesias manifest as at- and below-level neuropathic symptoms that are defined as either spontaneous (pain independent of peripheral stimuli) or evoked (occurring in responses to a noxious or non-noxious stimuli) [1, 3]. At-level pain is characterized by a dermatomal band of hypersensitivity within two segments adjacent to the site of injury [1, 5]. Most investigations into the development of at-level pain have focused on the spinal (lesion epicenter) and supraspinal mechanisms. However, recent evidence shows that injury to the cord results in enhanced intrinsic growth and hyperexcitability of adjacent peripheral afferents that may contribute to the development of at-level pain syndromes [6–9]. Furthermore, we recently demonstrated that quisqualic acid (QUIS) induced SCI results in DRG growth responses that are enhanced in animals that develop at-level dysesthesias, suggestive of a role in maladaptive peripheral growth in the development of post-spinal injury pain [6]. Peripheral nervous system contributions to SCI pain are supported by the finding that injury to the cord induces sprouting of primary afferent projections that may contribute to enhanced dorsal horn synaptogenesis and nociceptive signal amplification [10–15]. Despite the growing evidence to support maladaptive peripheral afferent structural plasticity in the development of at-level spinal injury pain, the intracellular signaling mechanisms that mediate these morphological effects are largely undefined.

A potential target mechanism impacting neuronal growth responses in post spinal injury pain involves the intracellular signaling molecule, glycogen synthase kinase-3β (GSK-3β). GSK-3β is highly expressed in the nervous system, and is particularly abundant in neurons where it plays a critical role in regulating neuronal growth, gene expression, and cell survival [16, 17]. Although GSK-3β exerts a diverse array of cellular functions, there is ample evidence for the influence of GSK-3β on neuronal plasticity through its regulation of structural proteins [18–22]. GSK-3β is serine/threonine kinase that under resting conditions is considered constitutively active, and is inactivated by phosphorylation of the Ser-9 residue. When active, GSK-3β causes growth cone collapse and suppresses axonal growth [20, 23, 24]. Extracellular growth promoting cues, such as neurotrophins and Wnts, signal to phosphorylate/inactivate GSK-3β, causing axon elongation [16, 20, 25–27]. Inactivation of GSK-3β allows the dephosphorylated GSK-3β substrates to bind cytoskeletal proteins to directly promote neurite outgrowth [20, 27–33]. In support of GSK-3β as a mediator of aberrant sensory growth and pain, GSK-3β is a convergent downstream signaling effector of established pro-nociceptive signals factors released following nervous system injury including nerve growth factor (NGF) and Wnts [34–39]. Injury induced upregulation of these chemical signals leads to activation of phosphatidylinositol 3-kinase (PI3K) and the subsequent inhibition of GSK-3β that may positively promote axonal elongation that contribute to the development of SCI pain [20, 40–42]. Although studies support a role for PI3K in the development of neuropathic and nociceptive pain following injury through neurochemical plasticity/central sensitization [36, 43–46], the role of PI3K-GSK3β signaling in modulating aberrant sensory growth and the development of spinal injury induced pain is unclear.

In this study, we investigated PI3K-GSK-3β signaling as a potential mechanism by which spinal injury promotes intrinsic growth of primary afferents that contributes to the development of at-level dysesthesias. Using the QUIS model of excitotoxic SCI, we demonstrated that injury to the dorsal gray matter was associated with GSK-3β inhibition and DRG growth responses associated with at-level dysesthesias. Further, PI3K mediated activation of GSK-3β reduced primary afferent growth responses and prevented the development of at-level dysesthesias.

## Results

### GSK-3β is inhibited in spinal dorsal horn and DRG following QUIS induced SCI

**Figure 1:**
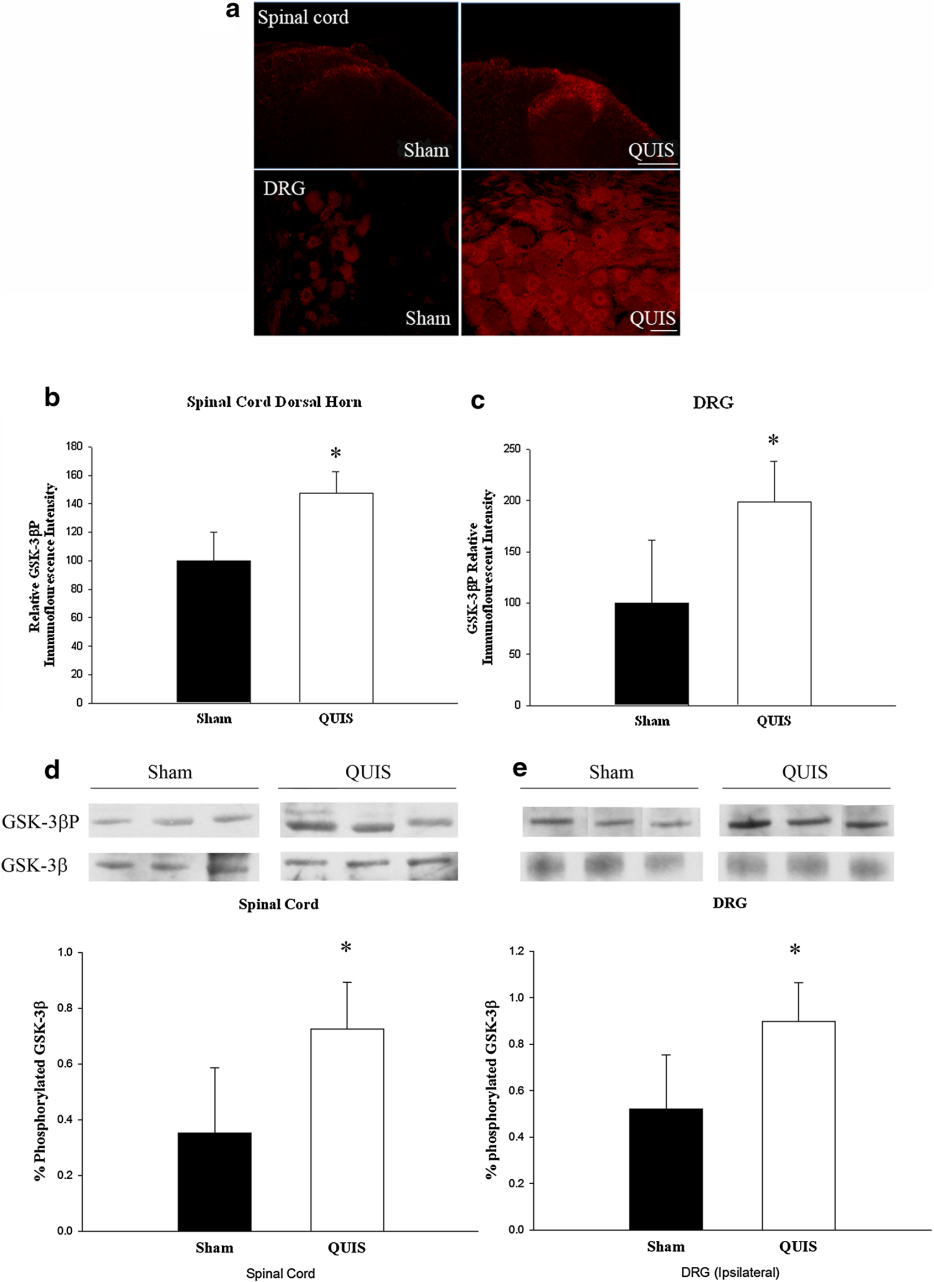
QUIS-injection increased GSK-3β^P^ in the spinal cord dorsal horn and DRG ipsilateral at and just caudal to the level of injury compared to saline-injected (sham) 14 days following injury. **a** Representative immunofluorescence image of GSK-3β^P^ in the superficial dorsal horn (L1) and DRG (L1, ipsilateral site of injection) shows increased GSK-3β phosphorylation (inhibition) following QUIS-injection compared to sham operated controls (Sham, n = 5, QUIS n = 6). **b**, **c** *Bar graphs* represent mean densitometric values of GSK-3β^P^ in spinal cord (**b**) and DRG (**c**). **d**, **e** Western blot analysis of the SC and DRG taken from the level of the lesion shows that GSK-3β^P^ expression was significantly increased 14 days following QUIS-injection (Sham, n = 3 and QUIS, n = 3). *Error bars* ± SEM (*p < 0.05). *Scale bars* spinal cord 100 μm, DRG 50 μm.

GSK-3β is constitutively active under resting conditions, with phosphorylation at Ser-9 residue resulting in GSK-3β inhibition and neurite elongation [19, 20, 47]. Our previous report demonstrated that QUIS-induced SCI resulted in enhanced DRG neurite outgrowth at 14 days post-injury [6]. To investigate the relationship of GSK-3β activity following QUIS induced injury we used a phosphor-Ser9-GSK-3β (GSK-3β^P^) antibody as a marker of inhibition and performed immunohistochemistry on spinal cord cross sections and DRG just caudal (L1) and ipsilateral to the site of injection 14 days after QUIS-injury or sham operation. Due to changes in laminae structures following QUIS injection, the L1 level was used for analysis. QUIS-injection (n = 6) resulted in significant increases in GSK-3β^P^ expression in the superficial dorsal horn and DRG compared to saline controls (n = 5) (Figure 1a), showing significant increases in thickness and intensity in GSK-3β^P^ expression that were uniformly distributed from the medial and lateral dorsal horn. Quantitative analysis showed that GSK-3β^P^ expression was significantly increased following QUIS-injection compared to sham injected-animals (p < 0.05). Western blot analysis of spinal cord and DRG (T12, the spinal injection level) 14 days following surgery, showed significant increases in ratios of GSK-3β^P^ over total GSK-3β in QUIS (n = 3) compared to sham-injected (n = 3) animals, providing further support of these biochemical changes following spinal injury (Figure 1d, e).

**Figure 2:**
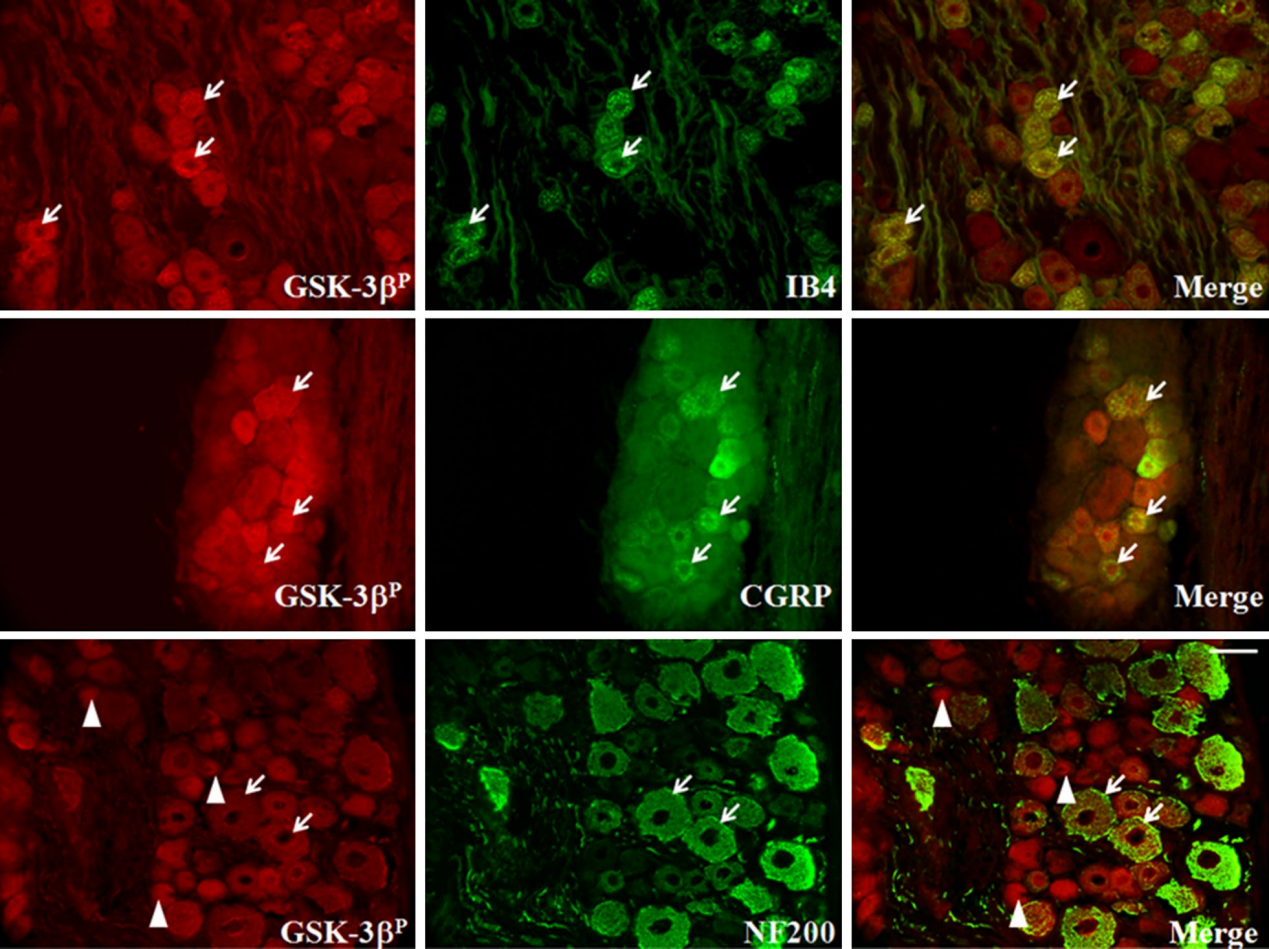
Double immuno-labeling with GSK-3β^P^ and nociceptive markers (IB4 and CGRP), and mechanoreceptive marker (NF200) from the level of QUIS-injection 14 days following SCI. *Merged images* demonstrate co-localization of nociceptive markers (afferent c-fibers) or mechanoreceptive markers (myelinated A-fibers) with GSK-3β^P^. *Yellow* regions indicate protein co-localization. GSK-3β^P^ was highly expressed in small-diameter, nociceptive neurons (arrowheads) and less frequent in large, mechanoreceptive fibers. *Scale bar* indicates 25 μm.

To determine the cell types that associated with GSK-3β^P^ expression following QUIS-injection, we double immunofluorescently labelled using phosphor-Ser9-GSK-3β with nociceptive markers (IB4, non-peptidertic; CGRP, peptidertic), and Aβ-fibers (NF200) (Figure 2). GSK-3β^P^ co-localized with IB4, CGRP and NF200, however expression was greatest with small nociceptive proteins. Together, these data support QUIS injection results in inhibition of GSK-3β in primary afferents (DRG and spinal cord afferent terminals) 14 days after injury.

### Intrathecal delivery of a PI3K inhibitor, LY294002, activates spinal GSK-3β and decreases QUIS spinal cord injury induced DRG growth responses

**Figure 3:**
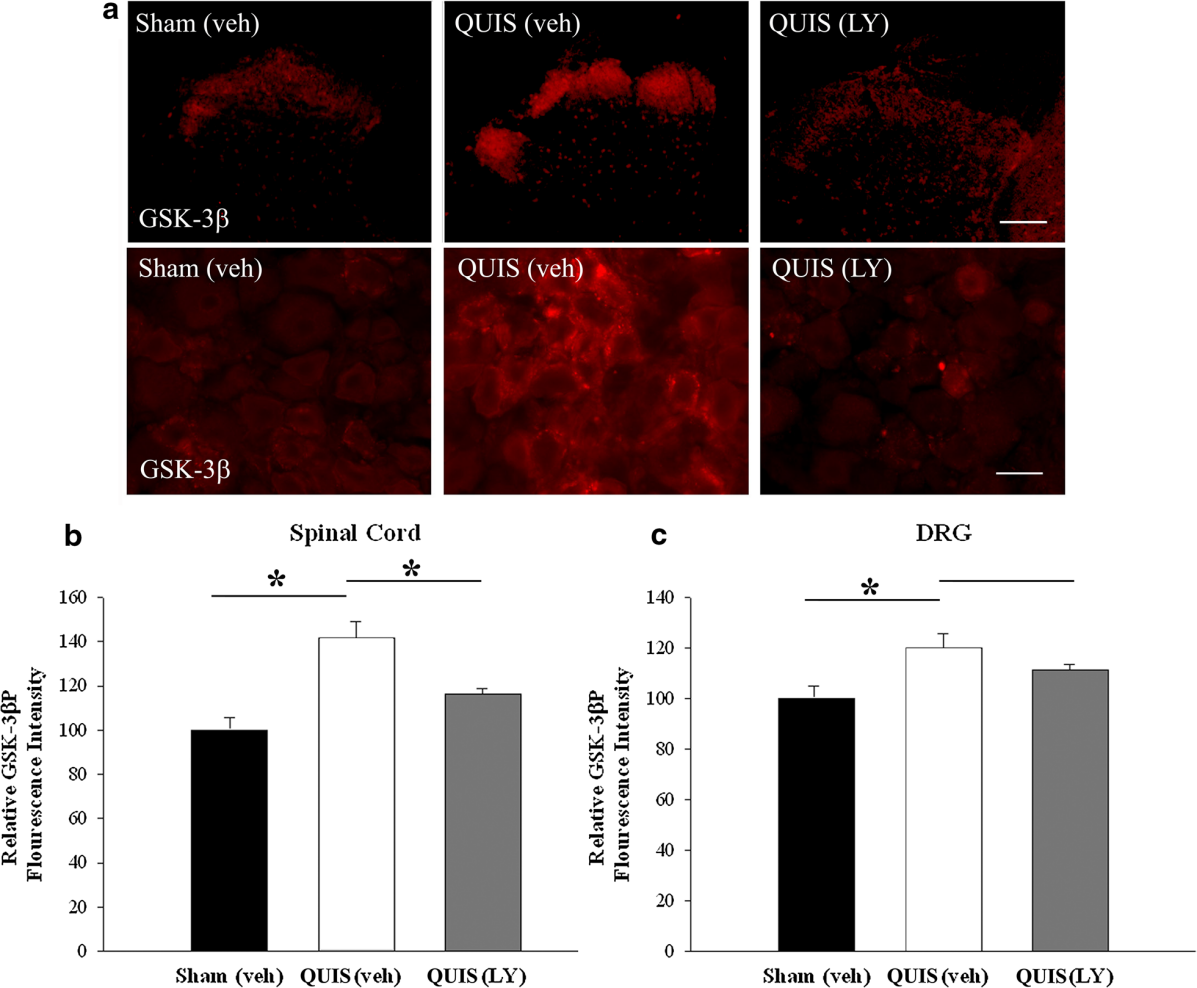
GSK-3β is inhibited in the spinal dorsal horn and DRG 3 day following QUIS injection. Immunofluorescent staining of GSK-3β^P^ in the spinal cord dorsal horn (*top row*) and DRG (*bottom row*) is increased 3 days following QUIS-induced SCI compared to sham vehicle treated controls; 3 days of LY294002 (LY) treatment significantly reduces GSK-3β^P^ staining following QUIS-induced SCI. Quantification of GSK-3β^P^ immunofluorescence intensity in SC (**a**) and DRG (**b**) (mean ± SEM. *p < 0.05. *Scale bar* 50 μm). Number of animals per group: sham vehicle (veh) n = 5, QUIS (veh) n = 5, QUIS (LY) n = 4.

We and others have recently reported that injury to the cord results in enhanced DRG growth responses, showing that SCI growth promoting effects are evident at early (3 days) and later stage (14 days) post injury [6, 8]. Since GSK-3β inhibition has an established role in neurite outgrowth [20, 22, 39], we asked if alterations in GSK-3β were evident early (3 days), and if application of a GSK-3β activator could reverse these spinal injury induced changes. For these experiments, rats were randomly assigned into three groups: sham vehicle (sham veh; 10% DMSO, n = 5) control, QUIS-vehicle (QUIS veh; 10% DMSO, n = 5), or QUIS LY294002 (QUIS LY, 2.5 μg/10 μL, n = 4), in each group animals received the same volume of infusions [36]. LY294002, a PI3K inhibitor and known activator of GSK-3β, was intrathecally delivered once daily for 3 consecutive days starting on the day of surgery. Three days following surgery, immunofluorescence staining was performed on DRG and spinal cord cross sections (from the level of L1-2, just caudal the lesion). Similar to later stage time point (14 days) sham vehicle treated animals showed low levels of GSK-3β^P^ expression (Figure 1), while QUIS-injury induced a significant increase in the ipsilateral spinal dorsal horn (p < 0.05) (Figure 3a–c). QUIS-injured animals treated with LY294002 showed a marked decrease in GSK-3β^P^ immunofluorescence in the spinal dorsal horn (p < 0.05) (Figure 3a–c). A similar response was observed in the DRG, however these results were not statistically significant (Figure 3a, c). Together, the results suggest that QUIS injection results in early (3 days) and extended (14 days) alterations in GSK-3β activity.

**Figure 4:**
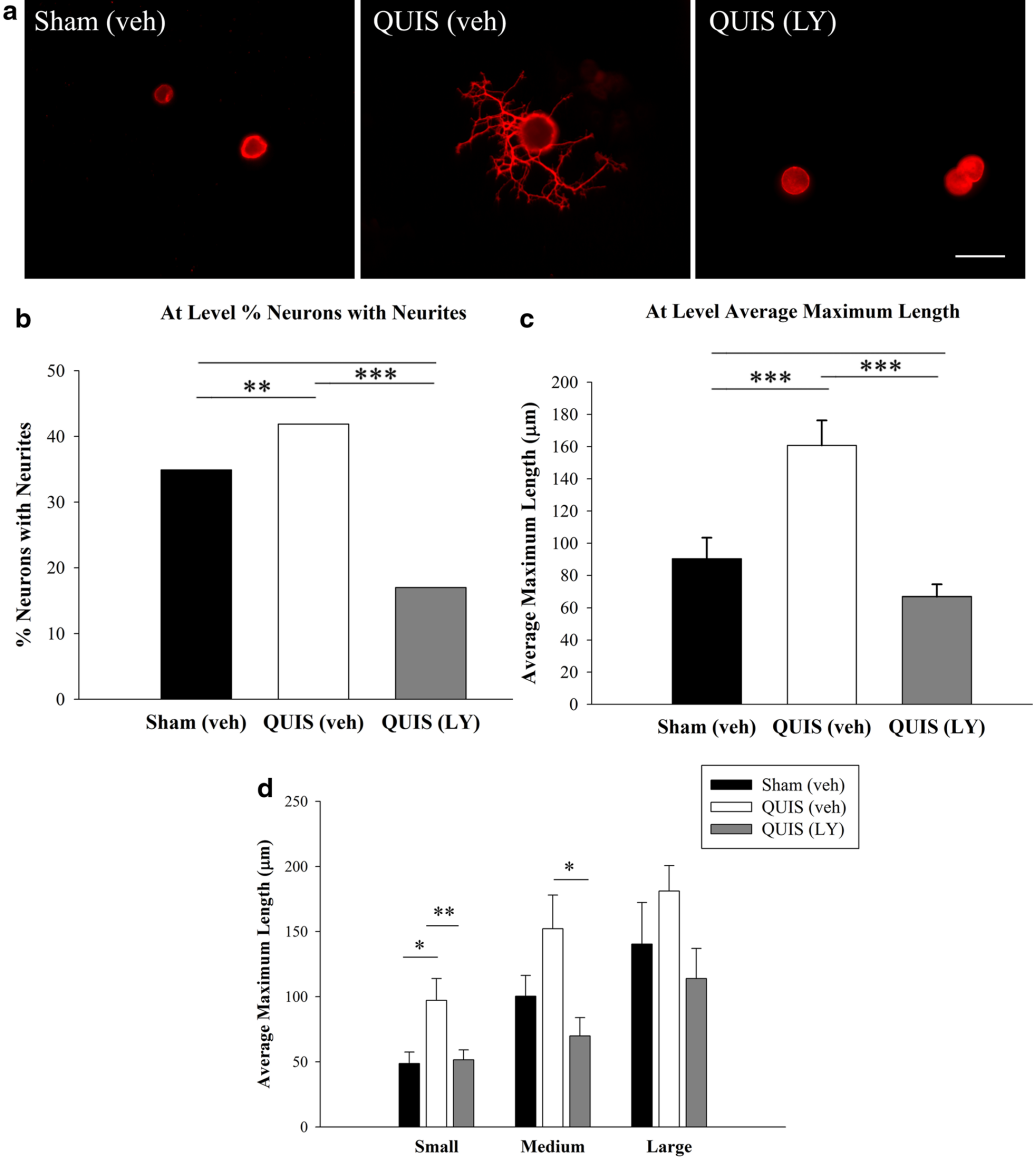
Intrathecal delivery of a GSK-3β activator (LY294002, LY; selective PI3K inhibitor) reduces DRG neurite outgrowth 3 days after QUIS-induced SCI. **a** QUIS-injection (QUIS veh) resulted in robust neurite outgrowth 3 days after injury compared to sham veh treated. Animals that received LY94002 following injury (QUIS LY) show growth responses similar to controls (sham veh). **b** Quantification of % of neurons with neurites. **c** Average length of longest neurite. **d** Mean length of small, medium, and large neurites. Number of animals per group: sham vehicle (veh) n = 5, QUIS (veh) n = 5, QUIS (LY) n = 4. Data are represented as mean ± SEM; *p < 0.05, **p < 0.01, ***p < 0.001. *Scale bar* 25 μm.

To determine if DRG neuronal growth paralleled the alterations in GSK-3β following QUIS injury, we assessed DRG growth responses three days following surgery and the effects of intrathecal drug LY294002 delivery. We found that QUIS-injection resulted in robust increases in the neurons with neurites (42%, n = neurons with neurites/neurons, 278/655, p < 0.01) and neurite elongation (160.7 μm ± 15.5, n = 278, p < 0.001) compared to sham-vehicle treated controls (35%, n = 243/696; 90.4 μm ± 13.0, n = 243) (Figure 4a–c). LY294002 delivered immediately following QUIS-injection and daily for the 3 days survival period, significantly reduced the injury induced DRG outgrowth (17%, n = 255/1,500; 70.2 μm ± 10.4, n = 255, p < 0.001) (Figure 4a–c). Although injury induced neurite elongation was evident in small, medium, and large neurons, only small neurons from QUIS-injected animals showed statistically significant increases compared to sham vehicle treated controls (97.2 μm ± 16.8, n = 79, p < 0.05) (Figure 4d). LY294002 attenuated these injury induced growth responses in small (19%, 51.5 μm ± 7.6, p < 0.01, n = 133) and medium (20%, 69.8 μm ± 14.2, p < 0.05, n = 68) neurons (Figure 4d). These data suggest that QUIS-injection results in early (3 days post injury) enhanced neuronal growth primarily in small fibers, and these effects can be blocked with LY294002 treatment.

LY294002 effect on reducing GSK-3β^P^ expression and potent effect on restricting early injury induced growth suggests that LY294002 may, in part, mediate its growth inhibiting effects through PI3K-GSK-3β signaling.

### Short term administration GSK-3β activator (LY294002) prevents the development of at-level spontaneous dysesthesias and reduces DRG outgrowth following QUIS spinal cord injury

**Figure 5:**
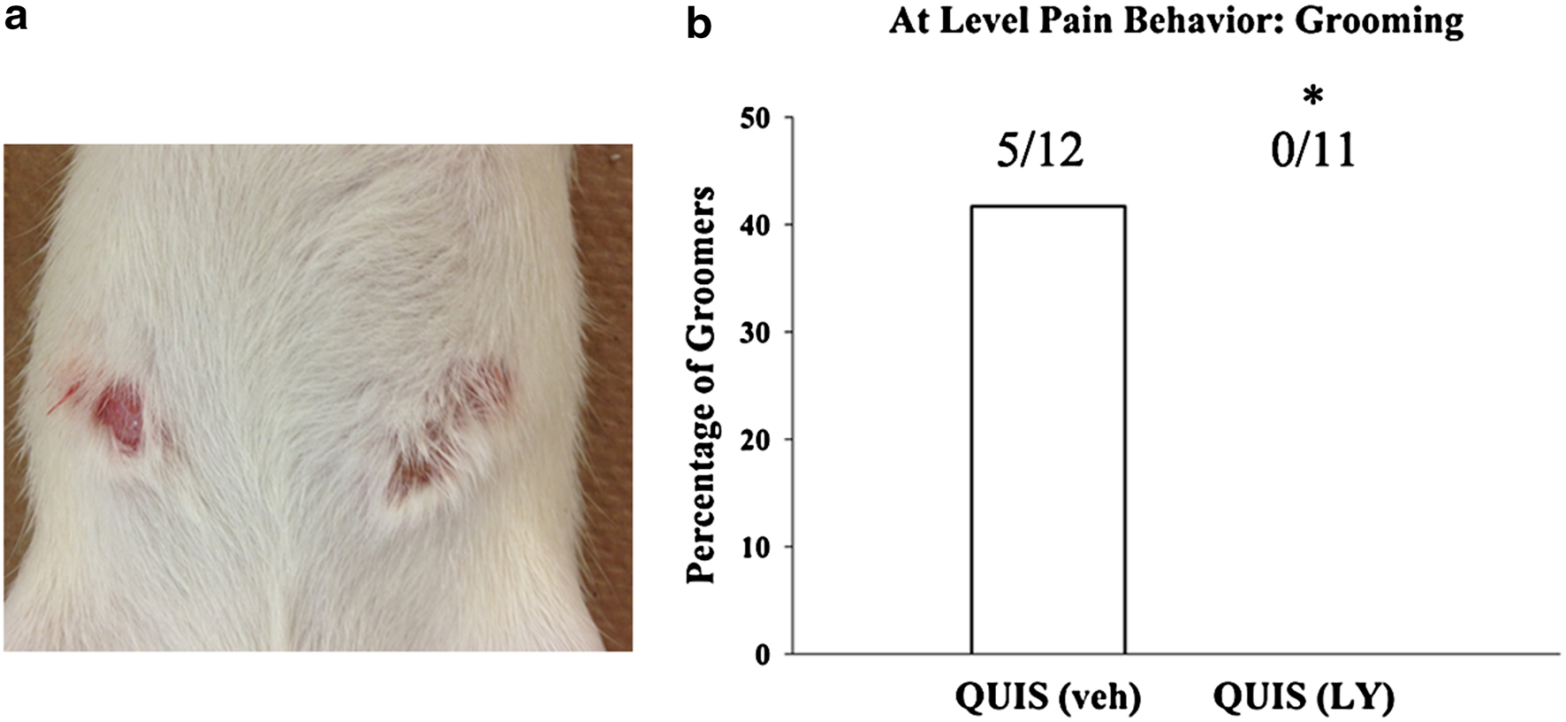
LY294002 treatment following QUIS-induced spinal injury prevents the development of at-level dysesthesias 14 days after injury. **a** Photograph of overgrooming lesion from QUIS vehicle treated animal primarily observed on at-level ipsilateral dermatome, class II bilateral groomer. **b** The incidence of overgrooming behavior was reduced from 42% (5/12) in animals treated with DMSO to 0% in animals treated with LY following SCI (0/11; p = 0.03).

The excitotoxic QUIS spinal cord injury model reliably results in the development of at-level dysesthesia characterized by excessive overgrooming behavior directed towards the dermatome associated with the level of injury that typically develops 6–12 days post injury [48, 49]. At-level dysesthesias develop 40–60% of all injured animals [48, 50]. This self-directed biting and scratching behavior results in hair removal and has been suggested to represent at-level sensory disturbances in this SCI model [48, 49] (Figure 5a). To assess the effect of LY294002 on at-level dysesthesias/overgrooming we extended the time course to 14 days survival following injury. Animals were randomly assigned the following groups: sham vehicle (sham veh; 10% DMSO, n = 9) control, QUIS-vehicle (QUIS veh; 10% DMSO, n = 12), or QUIS LY294002 (QUIS LY, 2.5 μg/10 μL, n = 11). Five QUIS veh animals developed overgrooming (class I, n = 2; class II, n = 3). LY294002 treatment initiated immediately following QUIS injury and once daily thereafter for three consecutive days significantly reduced the incidence of overgrooming behavior from 42% (n = 5/12) in QUIS veh animals to 0% (n = 0/11) in QUIS LY294002 treated animals (p < 0.05) (Figure 5b).

**Figure 6:**
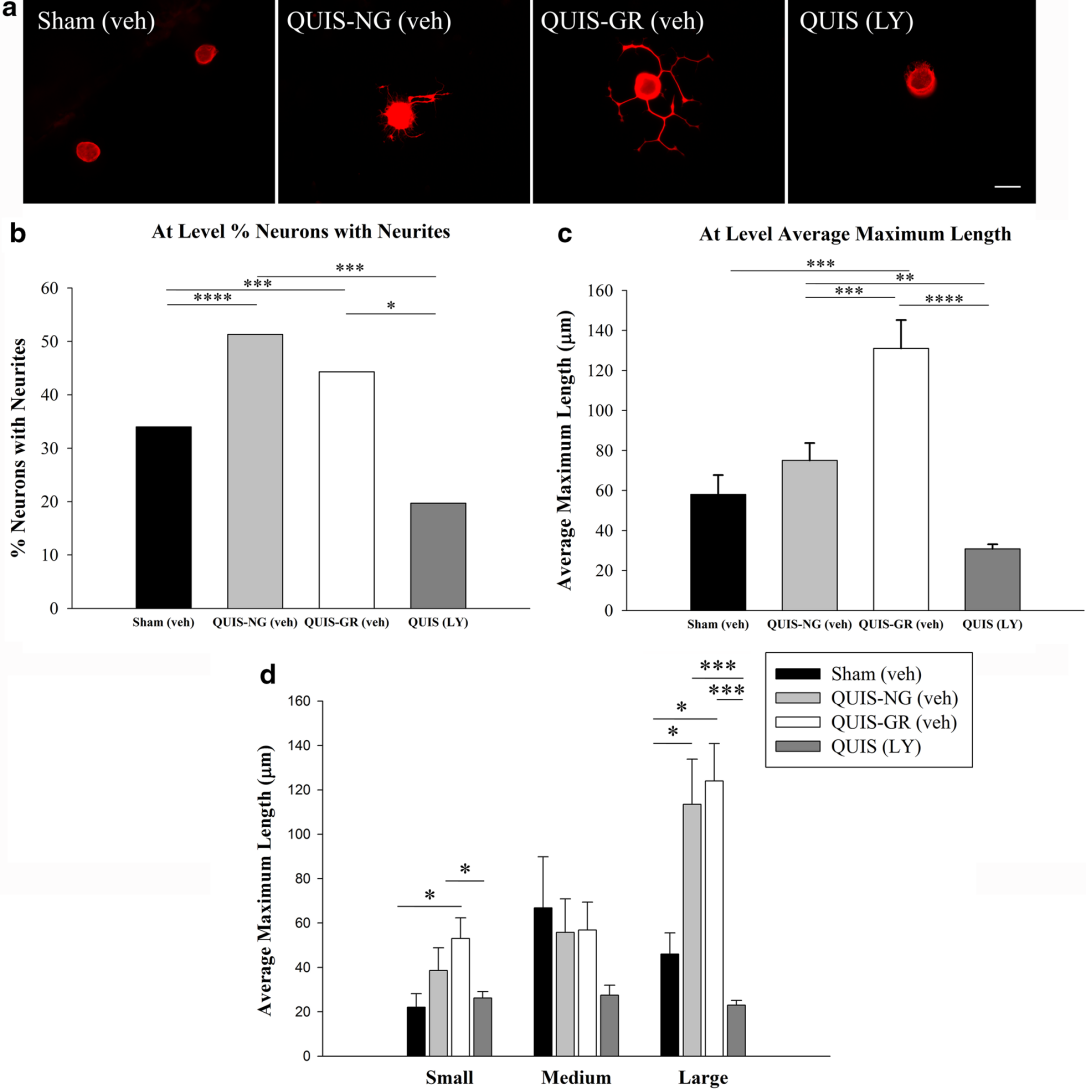
At-level QUIS-induced dysesthesias are associated with enhanced neurite outgrowth that is reduced with 3 day intrathecal delivery LY294002 (LY) 14 days following injury. **a** Representative immunofluorescent images of cultured DRG neurons from sham (sham veh, n = 9), QUIS nongroomers (QUIS-NG veh, n = 7), QUIS groomers (QUIS-GR veh, n = 5), QUIS LY294002 (QUIS LY, n = 11) taken ipsilateral to the injection site and grooming (T12) 14 days post-surgery. **b**, **c** DRGs from QUIS nongrooming (QUIS-NG veh) and grooming (QUIS-GR veh) animals show enhanced neurite growth initiation, DRG from QUIS-GR veh animals show enhanced neurite elongation compared to sham veh and QUIS-NG veh. Short term (3 days) intrathecal delivery of LY294002 (QUIS-LY) significantly decreased the percentage of neurons with neurites and the length of neurites following QUIS injury. **d** DRG growth responses were enhanced in small and large neurons 14 days post injury in QUIS-GR compared to sham veh controls. DRGs from QUIS-LY animals showed significant reductions in small and large DRG growth responses (mean ± SEM; *p < 0.05, **p < 0.01, ***p < 0.001, ****p < 0.0001).

To verify the LY294002 treatment effect on peripheral growth, cultured DRG were assessed from each of the following groups at the 14 day time point: sham vehicle (sham veh, n = 9), QUIS veh nongroomers (QUIS-NG veh, n = 12), QUIS veh Groomers (QUIS-GR veh, n = 5), and QUIS-LY294002 (QUIS LY, n = 11). Consistent with our previous report, cultured DRG from QUIS-GR-veh (44%, n = 524/1,184 neurons, p < 0.0001) and QUIS-NG veh (51%, n = 221/431, p < 0.001) animals showed an increase in the neurons with neurites compared to sham treated controls (34%, n = 144/423) (Figure 6a, b). QUIS-induced neurite elongation was most robust in animals displaying the sensory dysesthesias (QUIS-GR veh; 131.2 μm ± 14.2, n = 524) compared to sham (58.1 μm ± 9.7, n = 144, p < 0.001), QUIS-NG veh (75.0 μm ± 8.7, n = 221, p < 0.001) (Figure 6a, c). QUIS animals treated with LY294002 showed significant reductions in neurite formation (19.7%, n = 332/1,685, p < 0.05) and elongation (30.9 μm ± 2.2, n = 332, p < 0.0001) compared to the sham-vehicle animals (Figure 6b, c). The enhanced DRG growth from QUIS-Groomers was evident in small (53.0 μm ± 9.3, n = 159, p < 0.05) and large (113.5 μm ± 15.5, n = 236, p < 0.01), compared to sham (small, 22.1 μm ± 6.1, n = 35; large 46.1 μm ± 9.5, n = 78) (Figure 6d). QUIS induced growth of small and large DRG was significantly decreased with LY treatment (QUIS LY; small 26.2 μm ± 2.9, n = 62, p < 0.05; large, 23.0 μm ± 2.1, n = 151, p < 0.001) (Figure 6d). These results showed that at-level dysesthesias and enhanced DRG growth 14 days following QUIS spinal injury is prevented with early, short term LY294002 treatment.

## Discussion

This study demonstrates a role for altered GSK-3β activity resulting in enhanced DRG neurite outgrowth following excitotoxic spinal injury. Biochemical and immunohistochemical approaches showed that excitotoxic spinal lesions resulted in strong (early and persistent) GSK-3β inhibition in the spinal cord dorsal horn and adjacent DRG neurons. Intrathecal treatment with a PI3K inhibitor (LY294002), a known GSK-3β activator, blunted injury induced neurite initiation and elongation. Short term intrathecal delivery of LY294002, initiated at the time of injury, significantly reduced GSK-3β inhibition in the spinal dorsal horn and adjacent DRG and prevented the development of at-level dysesthesias. These data suggest that QUIS induced spinal injury results in GSK-3β inhibition and peripheral afferent growth that contributes to the development of at-level neuropathic pain following SCI.

### Peripheral growth responses in the development of at-level sensory disturbances

Historically, efforts to understand the development of neuropathic pain following SCI have focused on the injury epicenter and supraspinal regions [51]. This study along with previous results from our lab [6] has shown that QUIS spinal injury results in at-level induced dysesthesias associated with enhanced DRG growth. These finding are supported by Bedi et al. which showed similar at-level peripheral growth promoting effects along with spontaneous activity/hyperexcitability in a contusion model, suggesting that the growth responses were not unique to the SCI model [7, 8]. Consistent with these reports, we demonstrated that spinal injury induced DRG growth was evident early (3 days post-injury) and persisted throughout a 14 days survival period. Although latent DRG growth (14 day post-injury) was evident in small and large fibers, early injury induced growth (3 day post-injury) was most prominent in small DRG neurons, a finding that is supported by reports showing a temporal window of nociceptive sprouting occurring early post-SCI [8, 14]. It is possible that these growth promoting effects in small (C and Aδ) and large (Aβ) fibers contribute to the intraspinal sprouting and structural plasticity in dorsal horn circuitry that may, in part, contribute to pain post-SCI [11, 14, 15, 52–56]. These findings along with the increased GSK-3β^P^ expression (and expansion into deeper layers) in the dorsal horn, provide further evidence to support a role for maladaptive structural plasticity post-spinal injury. Collectively, the early growth of peripheral afferent post-spinal injury suggests that these responses may lead to maladaptive plasticity and reorganization in the dorsal horn afferents contributing to the development of at-level sensory disturbances.

### GSK-3β as a regulator of DRG outgrowth following SCI

These studies provide the first evidence to support a role for GSK-3β signaling as a regulator of peripheral afferent plasticity following spinal injury. GSK-3β is well-established for its role in neuronal morphogenesis via regulation of an extensive list protein substrates, such as collapsin response proteins [29, 32, 57], APC [20], CLASP-associated proteins [58, 59], δ-catenin [28, 30], microtubule associated proteins [60–64]. Regulation of these cytoskeletal proteins to promote neurite elongation is accomplished through fine-tuned control of GSK-3β activity [22, 39], were partial inhibition of GSK-3β via phosphorylation on Serine-9 leads to neurite elongation, and strong (inhibition) suppression or activation of GSK-3β leads to termination of neurite outgrowth. Here we demonstrated that QUIS induced SCI results in GSK-3β inhibition (spinal cord dorsal horn and adjacent DRG) and robust DRG neurite growth.

We demonstrated that opposing actions directed towards GSK-3β activation, using the PI3K inhibitor LY294002, effectively suppressed DRG neurite initiation and elongation following QUIS induced spinal injury. Although LY294002 is a potent inhibitor of PI3K/Akt pathways that targets GSK-3β activation, its effects are not direct; and therefore LY294002 may also activate Akt/mTOR pathways to restrict neurite outgrowth [20, 41, 65]. While LY294002 leads to activation of the mTOR pathway in the central nervous system, emerging evidence suggests that mTOR signaling does not control sensory afferent growth providing further evidence that PI3K-GSK-3β signaling is critical in mediating post injury DRG growth [42]. Our finding that intrathecally delivered LY294002 blocked the SCI induced increases in GSK-3β^P^ expression also supports that these effects are, in part, mediated through PI3K-GSK-3β signaling. This is in agreement with studies showing potent effects of LY294002 to activate GSK-3β and prevent neurite outgrowth [20, 41, 66]. Collectively, these data provide morphological evidence for the role of PI3K-GSK-3β signaling in SCI induced primary afferent growth.

### GSK-3β is a convergent signaling target to pro-nociceptive factors released in response to spinal injury

In support of GSK-3β as a mediator of aberrant afferent growth and sensory disturbances, GSK-3β is a convergent signaling effector of established pro-nociceptive signals including nerve growth factor (NGF), PI3K, and Wnts [26, 34, 35, 43, 45, 46, 67, 68]. Numerous studies have shown that peripheral and central nervous system injury results in upregulation of neurotrophins and Wnts that may inhibit GSK-3β and contribute the anatomical remodeling in spinal dorsal horn and DRG to induce neuropathic pain [34, 67, 69–71]. Furthermore, efforts to block the effects of NGF, Wnts, and PI3K are effective at preventing the development of neuropathic pain and autonomic dysreflexia [11, 34, 36, 45, 46, 69, 72]. Substantial evidence suggests that these extracellular signals released following injury converge to phosphorylate/inactivate GSK-3β to cause neurite elongation [20, 26, 73–77]. Interestingly, studies have demonstrated that activation of PI3K is required for NGF-induced neurite elongation, and therefore our finding that demonstrated alterations PI3K-GSK-3β signaling following QUIS spinal cord injury provides an intracellular link that underlies these structural responses following injury [20, 78, 79].

### Altered GSK-3β activity in the spinal dorsal horn and DRG in neuropathic pain

Recent studies have provided evidence for the role of altered GSK-3β activity in development of neuropathic pain, showing that GSK-3β^P^ (inactive) is distributed in the spinal cord dorsal horn axons where it is positioned to influence sensory growth and synaptic plasticity [66, 80]. Although the temporal relationship between GSK-3β activities and pain following nervous system injury are largely unexplored, Weng et al. recently showed that peripheral nerve injury resulted in early increases in GSK-3β^P^ in the spinal cord dorsal horn [80]. This finding is consistent with our data (Figure 1, 3) showing early and persistent inhibition of GSK-3β in the DRG and spinal cord dorsal horn following excitotoxic SCI (evident at 3 and 14 days post-injury). In support of these findings, peripheral nerve injury induces early activation PI3K-Akt in spinal dorsal horn and DRG neurons, an established upstream signal to inhibit GSK-3β activity [36]. Furthermore, activation of PI3K/Akt following peripheral nervous system injury are strongly expressed in small, nociceptive neurons that have an established role in early central sensitization which contributes to the development of neuropathic pain [36, 43, 81]. Interestingly, reports have shown that activation of PI3K-GSK-3β following injury are transient, occurring within 3 days post-injury [36, 80]. Our reported inhibition of GSK-3β that extended throughout 14 days post-injury may be attributed to differences between peripheral vs. central injury, as well as the progressive nature created by the QUIS-excitotoxic spinal injury [48]. Additional evidence for the role of GSK-3β in SCI pain is supported by the finding that NGF, an inhibitor of GSK-3β with a pivotal role in primary afferent sprouting, is increased in the spinal cord and DRG following SCI [20, 25, 35, 67]. Further studies are needed to examine how different SCI models impact GSK-3β, activity as well as the time course for these changes. Nonetheless, our studies provide the first link by which spinal injury can directly impact GSK-3β activity in a manner that would lead to sensory afferent outgrowth/sprouting and sensory dysesthesias.

Although GSK-3β plays a pivotal role in regulating structural neuronal plasticity [20, 22, 27, 79], GSK-3β also regulates other cellular functions including cell survival pathways, and more recent reports demonstrating a role in pro-inflammatory processes [16, 19, 80, 82]. As such, activation of GSK-3β may contribute to enhanced secondary damage and neuronal apoptosis associated with SCI [83]. To ensure that our observed differences with LY294002 treatment were not related to enhanced neuronal death, cell quantification of the proportion of different neuronal types and cell densities showed that the overall number and distribution of cell size of our neuronal cultures from LY294002 treated were comparable to those from sham-operated and QUIS-injected animals (data not shown) [6, 8], suggesting that our reported differences were not related to enhanced neuronal death from drug treatment. Furthermore, there are emerging reports showing that application of GSK-3β inhibitors may prevent the development of pain or reduce secondary damage following peripheral or central nervous system injury potentially through reduced inflammatory responses [83–85]. Several of these studies employ preemptive treatments [80, 84, 85], strategies that may have limited efficacy for the treatment of neuropathic pain following SCI resulting from unexpected trauma or ischemia. Other studies use systemic (intraperitoneal) drug delivery which lacks targeted delivery and may, therefore, have off target consequences that contribute to the differences observed with activation vs. inhibitory action of GSK-3β to prevent neuropathic pain [83, 86]. It is plausible that the reported protective effects of both GSK-3β inhibition and activation may be related to different mechanism of action based on mode of delivery (intraperitoneal vs. intrathecal), pain model (acute vs. neuropathic), and the timing of drug administration (preemptive vs. acute short term). Our study provides evidence into the temporal and spatial alterations of GSK-3β activity following spinal injury; to establish that early intratehecal delivery of LY294002 (a known GSK-3β activator) was successful at preventing the development of at-level dysesthesias.

### PI3K-GSK-3β signaling in sensory and motor neurite outgrowth

Molecular signaling pathways, such as PI3K-GSK-3β that control axonal growth are shared between sensory and motor fibers [20, 25], making therapeutics that regulate structural plasticity challenging. The growth promoting effects of GSK-3β has made inhibition of this enzyme an appealing target to promote motor fiber regeneration following SCI. This has resulted in clinical and experimental studies using GSK-3β inhibitors to promote axonal regeneration following SCI [25, 87, 88]. Recent studies now raise the possibility that GSK-3β inhibition will not effectively promote long-distance axonal growth and may further restrict spinal cord regeneration [32, 87, 89]. Here we also demonstrated that excitotoxic SCI resulted in GSK-3β inhibition, and further suppression with GSK-3β inhibitors may prevent sensory outgrowth and possibly the desired axonal motor fiber regeneration.

Understanding of the temporal and spatial nature of the growth promoting effects are of particular importance following SCI, where efforts to control structural neuroplasticity and regeneration are opposing, to promote motor fiber regeneration and yet limit sensory sprouting and growth that contributes to pain syndromes post-injury [52, 53]. Our finding that DRG growth responses contribute to at-level pain syndromes is significant as therapeutics to prevent maladaptive growth responses work towards targeted approaches to improve clinical efficacy [52, 53, 90, 91]. Our short term intrathecal delivery of LY294002 was effective at blocking peripheral outgrowth and at-level dysesthesias, however future studies investigating targeted delivery to the periphery (DRG) are needed to prevent pain and allow for motor fiber recovery post-SCI. Furthermore, our previous report and other studies have demonstrated that SCI results in primary afferent growth and sensitization in segments remote from the site of injury [6, 8, 14, 15, 92]. Studies are underway to investigate the effect of injury induced growth on distant segments to determine if activation of GSK-3β can prevent and reverse the development of below-level hyperalgesia and allodynia following SCI [48]. This study identified a therapeutic window specific to target inhibition of at-level sensory growth. Future studies using traumatic SCI models to incorporate the motor complications and determine the appropriate therapeutic timeline to maximize motor recovery while minimizing the risk for the development of pain are needed.

## Conclusion

In summary, these studies identify PI3K-GSK-3β signaling as a mechanism controlling maladaptive primary afferent growth in the development of at-level excitotoxic SCI induced dysesthesias. We have shown that acute short term (3 days) treatment with a GSK-3β activator, LY294002, is sufficient to prevent the development of at-level sensory dysesthesias and reverse injury induced DRG growth responses. These data support that targeting GSK-3β may be therapeutic to prevent maladaptive growth and at-level pain following SCI.

## Methods

### Animals and excitotoxic QUIS induced spinal cord (dorsal horn) injury

All experimental procedures were approved by the Institutional Animal Care and Use Committee at East Carolina University. The surgical intramedullary injection technique was similar to previously described methods [48, 93]. Briefly, male Long Evans rats (175–200 g) were anesthetized, a posterior midline incision was made along the thoracolumbar junction. A laminectomy was completed at approximately T11-L1 and dura was incised longitudinally and retracted. A glass micropipette with 5–10 μm tip diameter was attached to a 10 μm Hamilton syringe for injections. The syringe was mounted on a microinjector (Kopf 5000) and micromanipulator. Intramedullay injections of 125 mM quisqualic acid (QUIS, Sigma, St. Louis, MO, USA) were made in the dorsal root entry zone of the spinal cord (between the midline vessel and lateral aspect) at a depth of 1,000 μm below the surface of the cord. Unilateral injections were made in a single segment at the T12 spinal level. Animals received either 1.2 μL of phosphate buffered saline (PBS, sham) or QUIS over a 60-s time interval (3 tracks of 0.4 μL separated by 0.3 μm parallel to the long axis of the cord). In an initial set of experiments, animals underwent QUIS-injection or sham-injection and were allowed to survive for 14 days following surgery without cannulation or drug delivery (sham, n = 5; QUIS, n = 6).

### Surgical procedures for intrathecal drug delivery

For animals receiving intrathecal injections, immediately following QUIS or PBS spinal injections, a polyethylene catheter (PE-10 tubing) was inserted under the open dura into the intrathecal space immediately caudal to the site of injection. The catheter was secured in place by suturing to spinal muscles along the length of the surgical incision. The rostral end of the catheter was tunneled under the skin, externalized at the back of the neck and secured with sutures and skin adhesive (VetBond). Spinal muscles were closed around the catheter, and the incisions were closed with staples. Animals from each group (QUIS-injected vs. sham-injected) were randomized to receive intrathecal LY294002 (PI3K inhibitor, 0.5 µg in 10 µL of vehicle) or an equal volume of vehicle (10% DMSO). Drugs were delivered once daily for 3 days, and animals were euthanized at 3 or 14 days following surgery. The number of animals for each time point were as follows: 3 day Sham vehicle (veh) n = 5, QUIS (veh) n = 5, QUIS (LY) n = 4; QUIS (LY) n = 3; 14 day Sham (veh) n = 9, QUIS (veh) n = 12, QUIS (LY) n = 11.

### DRG cultures and morphological analysis

Culturing of DRG were performed as previously described [94]. At the end of the survival period (3 or 14 days), animals were deeply anesthetized with isoflurane while the thoracolumbar vertebral column was removed. Two DRG were collected ipsilateral to the injection and side of grooming (T11-T12) in QUIS and sham (saline-injected) animals and immediately placed in Hibernate A (BrainBits, Springfield, IL, USA) with 10% horse serum and 100 microgram/μL penicillin, 100 μg/μL streptomycin. DRG were washed in plating media containing Pen/Strep, disassociated enzymatically via use of (2 μg/mL) collagenase (Sigma, St. Louis, MO, USA) and 0.25% trypsin/EDTA (Invitrogen, Grand Island, NY, USA) and mechanically via microsnipping and trituration through a polished Pasteur pipette. Cells were plated at low density onto glass coverslips in 24-well plate (coated with 100 μg/μL of poly-l-lysine and 10 μg/μL laminin), incubated at 37°C in plating medium containing DMEM/F12 containing 10% fetal bovine serum, 10% horse serum, 2 mM glutamine, 100 μg/μL penicillin, 100 μg/μL streptomycin. After 24 h cells were fixed with 4% paraformaldehyde and washed with PBS. Cells were permeabilized with 0.2% Triton, blocked with 10% PBS, and stained with rabbit anti-tubulin III (Sigma, St. Louis, MO, USA, 1:75) antibody conjugated to anti-rabbit secondary cy3 (Jackson ImmunoResearch, 1:300). Coverslips were mounted onto slides using ProLong Gold with DAPI (Invitrogen), used to visualize the cell nucleus. Images were captured at 40× magnification with the Leica DM4000 microscope using a Q imaging Retiga 2000R camera. Image-Pro Express 6.3 software was used for neuronal measurements. Soma size was measured and cells were categorized based on the following criteria: small <30.4 μm, medium 30.5 - 45.4 μm, and large >45.0 μm. Neurons with neurites above 10 μm were measured for the length of the longest neurite. The total number of neurons, total number of neurons with neurites (above 10 μm), and average maximum neurite length were calculated for each condition. Measurements for longest neurite were traced from the soma to the most distal tip of the neurite. DRG from the respective groups (3 days: sham veh, QUIS veh, QUIS LY; 14 days sham veh, QUIS-NG veh, QUIS-GR veh, and QUIS-LY) were pooled a minimum of five coverslips and more 400 neurons were counted for each condition. An equal number of cells were plated for each condition, cell densities 1 day after dissociation was 80 ± 17 cells/coverslip. Experiments were performed by an investigator that was blinded to the conditions.

### Western blotting

Spinal cord from T12 (at-level injection) and adjacent DRG from saline (sham) (n = 3) and QUIS-injected (n = 3) animals (14 day survival period) were homogenized using RIPA buffer (150 mM NaCl, 10 mM HEPES pH 7.3, 2 mM EDTA, 0.2% SDS, 0.5% sodium deoxycholate, 1% Triton X-100) with protease inhibitors (Roche Complete Protease Inhibitor, Indianapolis, IN, USA). Samples were centrifuged at 14,000 g, supernatant was collected and protein concentration was determined by Pierce BCA assay (Thermo Scientific). Samples were boiled for 5 min and equal amounts of protein were loaded and run on SDS-PAGE gels, transferred to nitrocellose membranes, and immunoblotted for total anti-mouse GSK-3β and anti-rabbit phospho-Ser9-GSK-3β (GSK-3β^P^) (Cell Signaling Technology, Beverly, MA; 1:1,000). Bound antibodies were detected with horseradish peroxidase-conjugated anti-rabbit and anti-mouse immunoglobulin G (Promega, Madison, WI, USA; 1:2,500). Western-blotted protein bands were quantified with UN-SCAN-IT software (Silk Scientific, Inc. Orem, UT, USA). Proteins levels reported in figures were obtained as a ratio between GSK-3β^P^ and total GSK-3β.

### Immunohistochemistry

Spinal column and dorsal root ganglia were removed and placed in 4% paraformaldehyde for 24 h and transferred 30% sucrose in phosphate buffered saline (PBS). Spinal cord (L1 spinal level, 25 μm) and DRG (L1 ipsilateral lesion, 16 µm) were cryosectioned using a Leica 2400 sledge microtome. Tissue sections were blocked (3% donkey serum, 1% BSA, 0.3% Triton X-100 in PBS) for 1 h at room temperature. Tissue was then incubated with primary antibody (GSK3-β^P^, 1:50, Cell Signaling Technology) and placed in a humidity chamber overnight at 4 °C. Double immunolabeling of DRG sections were incubated with GSK-3β^P^ and nociceptive markers: Isolectin-B4 20 μg/ml; (Sigma, St. Louis, MO, USA), calcitonin gene related peptide (CGRP; 1:150 abcam, Cambridge, MA, USA), and myelinated A-fiber marker neurofilament-200 (NF200; 1:200 abcam, Cambridge, MA, USA). Tissue was rinsed with PBS and incubated with secondary antibody for 1 h at room temperature (rabbit cy3, 1:300 and for double labeling combined with mouse-FITC (1:100) (Jackson ImmunoResearch, West Grove, PA, USA). Sections were rinsed with PBS 3 times and coversliped with VectaShield with DAPI (Burlingame, CA, USA).

### Immunohistochemistry image analysis

Spinal cord dorsal horn (L1) and DRG (L1) images were captured <24 h after coverslipping using a Leica DM4000 microscope, images were acquired using a Retiga 2000R camera (Q imaging) on a at 10× (SC) and 40× (DRG) magnification. For each animal, every third or fourth of consecutive sections. (4 DRG and 6 dorsal horn images) were captured at the same magnification, lens aperture and exposure time using Image Pro Express software (Media Cybernetics, Rockville, MD, USA). Image analysis was performed using ImageJ (NIH). Due to changes in laminae structure following QUIS injection, we assessed changes in immunofluorescence density using segments just caudal to the site of injury (L1) using an area of interest (AOI) method previously described [95]. For dorsal horn analysis, the AOI was placed in the most central portion horizontally with the top corners of the box at the most superficial edge of the lamina. Background density threshold was established for each image using the standardized box (AOI). Immunofluorescence for dorsal horn laminae were measured and subtracted from background densities. An average optical density was obtained for each animal and a mean value for each group was reported. A percent change from sham to QUIS-injected animal was determined in order to show differences in the two conditions. All histological analysis was performed by a blinded investigator.

### Excessive grooming behavior

Animals were assessed once daily starting on day 6 post-injection for the presence of overgrooming (at-level spontaneous dysesthesia) directed towards the dermatome associated with the level of injury [48, 96]. This behavior is characterized by self-directed biting/scratching that results in hair removal and progressive damage to the skin and has been suggested to represent at-level spontaneous sensory disturbances in this model [48]. The excessive grooming behavior was categorized as class I–IV as previously described [97]: class 1—hair removal over portions of a dermatome, class II—extensive hair removal with signs of damage to the superficial skin layers, class III—hair removal and damage to dermal layers of the skin, class IV—damage to subcutaneous tissue.

### Statistical analysis

Statistical analyses were performed using GraphPad Prism 5.04 software (San Diego, CA, USA). All data are reported as mean ± standard error (SEM) throughout, with statistical significance set at p < 0.05. One way ANOVA followed by Bonferron’t post hoc comparison or paired t tests when applicable were used to identify differences between group means. Non parametric values were analyzed with Pearson Chi square test.

## Abbreviations

CGRP: calcitonin gene-related peptide
DRG: dorsal root ganglia
DMSO: dimethyl sulfoxide
QUIS: quisqualic acid
GSK-3β: glycogen synthase kinase 3 beta
LY: LY294002
PI3K: phosphoinositide 3-kinae
SCI: spinal cord injury
veh: vehicle
